# Adipokines secretion in feline primary adipose tissue culture in response to dietary fatty acids

**DOI:** 10.1186/s12917-019-2065-8

**Published:** 2019-09-06

**Authors:** M. Mazaki-Tovi, S. R. Bolin, P. A. Schenck

**Affiliations:** 1 Department of Pathobiology and Diagnostic Investigation, Diagnostic Center for Population and Animal Health College of Veterinary Medicine, Michigan State University, East Lansing, MI 48824 USA; 2 Present address: Hebrew University Veterinary Teaching Hospital, Koret School of Veterinary Medicine, The Hebrew University of Jerusalem, P.O. Box 12, 76100 Rehovot, Israel; 3 Present address: Veterinary Consulting, Dewitt, MI 48820 USA

**Keywords:** Adipogenesis, Cytokines, N-3 fatty acids, N-6 fatty acids, Saturated fatty acids

## Abstract

**Background:**

Obesity in cats has been associated with alterations in adipokines including: adiponectin, interleukin-6 (IL6), and tumor necrosis factor-α (TNFα). Omega-3 polyunsaturated fatty acids have multiple beneficial effects on obesity-associated disorders, and therefore may alleviate these alterations. This study aimed to determine the effects of body condition, fat depot, troglitazone, and different fatty acids on secretion of adiponectin, IL6 and TNFα from adipose tissue of healthy cats. Subcutaneous and visceral adipose tissue samples were collected from 18 healthy intact female cats, and body condition score (Range 3–7/9) was determined. Concentrations of adiponectin were measured in mature adipocytes cultures and concentrations of IL6 and TNFα were measured in stromovascular cells cultures following treatment with control medium, troglitazone at 10 μM, eicosapentaenoic acid, arachidonic acid, or palmitic acid, at 25, 50, or 100 μM.

**Results:**

Stromovascular cells of visceral origin secreted higher concentrations of IL6 than corresponding cells of subcutaneous origin (*P* = 0.003). Arachidonic acid treatment at 25, 50, and 100 μM increased IL6 secretion in subcutaneous (*P* = 0.045, *P* = 0.002, and *P* < 0.001, respectively) and visceral (*P* = 0.034, *P* = 0.001, and *P* < 0.001, respectively) stromovascular cells. Eicosapentaenoic acid treatment increased TNFα secretion in subcutaneous stromovascular cells at 25, 50, and 100 μM (*P* = 0.002, *P* = 0.001, and *P* = 0.015, respectively) and in visceral stromovascular cells at 50 μM (*P* < 0.001). No significant effect on medium adiponectin concentration was observed following troglitazone treatment (*P* = 0.4) or fatty acids treatments at 25 (*P* = 0.2), 50 (*P* = 0.8), or 100 (*P* = 0.7) μM. Body condition score did not have significant effects on medium concentrations of adiponectin (*P* = 0.4), IL6 (*P* = 0.1), or TNFα (*P* = 0.8).

**Conclusions:**

This study demonstrated higher basal secretion of IL6 from visceral compared to subcutaneous adipose tissue, a stimulatory effect of arachidonic acid on secretion of IL6 and a stimulatory effect of eicosapentaenoic acid on TNFα from feline adipose tissue.

## Background

Adipose tissue has an important role in energy homeostasis by serving as a storage organ as well as through secretion of adipokines, including adiponectin, interleukin-6 (IL6), and tumor necrosis factor-α (TNFα). It is a heterogeneous tissue comprised of various types of cells, including mature adipocytes and stromovascular cells (SVC, preadipocytes, fibroblasts and undifferentiated mesenchymal stem cells, endothelial cells, and macrophages). Adiponectin is synthesized and secreted predominantly by mature adipocytes [1]. It has profound effects to increase insulin sensitivity and decrease lipid concentrations [2] as well as anti-inflammatory and anti-atherosclerotic properties [3, 4]. Interleukin-6 and TNFα are produced by the SVC [5, 6], mainly the macrophages [7].These pro-inflammatory cytokines also display multiple effects on carbohydrate and lipid metabolism, leading to impairment of insulin sensitivity [8] and accelerated lipolysis [9].

The nuclear receptor peroxisome proliferator-activated receptor γ (PPARγ) is expressed predominantly in the adipose tissue, where it has an important role in glucose and lipid homeostasis [10], and also in macrophages, where it inhibits cellular activation [11]. In cats, expression of PPARγ mRNA was demonstrated in adipose tissue [12] and pharmacological agonists were shown to improve insulin sensitivity and lipid metabolism [13, 14], and to increase adiponectin concentrations [14]. In addition, agonists of PPARγ have been shown to increase circulating adiponectin concentrations and to decrease TNFα concentrations in diabetic human patients [15, 16].

Obesity in cats, similar to other species, has been associated with decreased adipose tissue mRNA expression and circulating concentrations of adiponectin as well as increased adipose tissue expression of TNFα and IL6 [17–20]. Low adiponectin concentrations and systemic low-grade inflammation have been suggested in the pathogenesis of several obesity-associated metabolic disorders in humans, including insulin resistance, hepatic steatosis, dyslipidemia, and atherosclerosis [4, 21, 22]. In cats, insulin resistance [17] and diabetes mellitus [23] were associated with decreased adiponectin concentrations, whereas hepatic lipidosis [24] was associated with increased adiponectin concentrations that were suggested to be related to hepatic injury, rather than lipidosis specifically.

Omega-3 polyunsaturated fatty acids (n3PUFA) have multiple beneficial effects on obesity-associated disorders in humans and animals. Feeding an n3PUFA rich diet to obese cats resulted in improved insulin-sensitivity compared to a diet rich in saturated fatty acids (SFA) [25]. In humans, in addition to an insulin-sensitizing effect, n3PUFA have been shown to have cardioprotective, hypolipidemic, and anti-inflammatory effects [26–29].

In view of the beneficial effects of n3PUFA in disorders that are associated with decreased concentration of adiponectin and increased concentrations of IL6 and TNFα, it is possible that these alterations may be ameliorated by n3PUFA. The aim of this study was to determine the effect of fat depot, body condition and fatty acids (FA) on secretion of adiponectin, IL6, and TNFα from feline adipose tissue.

## Results

### Cats and cultures

All cats were mixed breed. Age was estimated to range from 6 to18 months. Body condition score (BCS) ranged from 3 to 7 (Median: 5). BCS distribution was as follows: 3 (*n* = 1), 5 (*n* = 9), 6 (*n* = 7), 7 (*n* = 1).

The packed adipocytes volume (PAV) ranged from 2 to 8% (median: 5%). No significant effect of PAV on medium adiponectin concentrations was demonstrated in the analysis of the effect of BCS, fat depot and troglitazone treatment (*P* = 0.3) or the analyses of the effect of fatty acids (FA) treatment at 25 μM (*P* = 0.9), 50 μM (*P* = 0.3) or 100 μM (*P* = 1.0).

The SVC score ranged from 1 to 5 (median: 4). A significant effect of SVC score on medium IL6 concentrations was demonstrated in the analysis of the effect of BCS and fat depot (*P* = 0.001), and in the analyses of the effect of FA treatment at 25 μM (*P* = 0.005), 50 μM (*P* < 0.001), and 100 μM (*P* < 0.001). An increase of 1 unit in SVC score was associated with an increase of 3.2 ng/mL (1.3–5.1), 4.4 ng/mL (1.4–7.4), 5.2 ng/mL (2.3–8.2), and 4.7 ng/mL (2.2–7.1) in adjusted IL6 concentrations in the analysis of BCS and fat depot, and at 25 μM, 50 μM, and 100 μM FA treatment, respectively. No significant effects of SVC score on medium TNFα concentrations were demonstrated in the analysis of the effect of BCS and fat depot (*P* = 0.6) or the analyses of the effect of FA treatment at 25 μM (*P* = 0.2), 50 μM (*P* = 0.4) and 100 μM (*P* = 0.8).

### Effect of BCS and fat depot on adipokines

BCS did not have significant effects on medium concentrations of adiponectin (*P* = 0.4), IL6 (*P* = 0.1), or TNFα (*P* = 0.8).

**Fig. 1:**
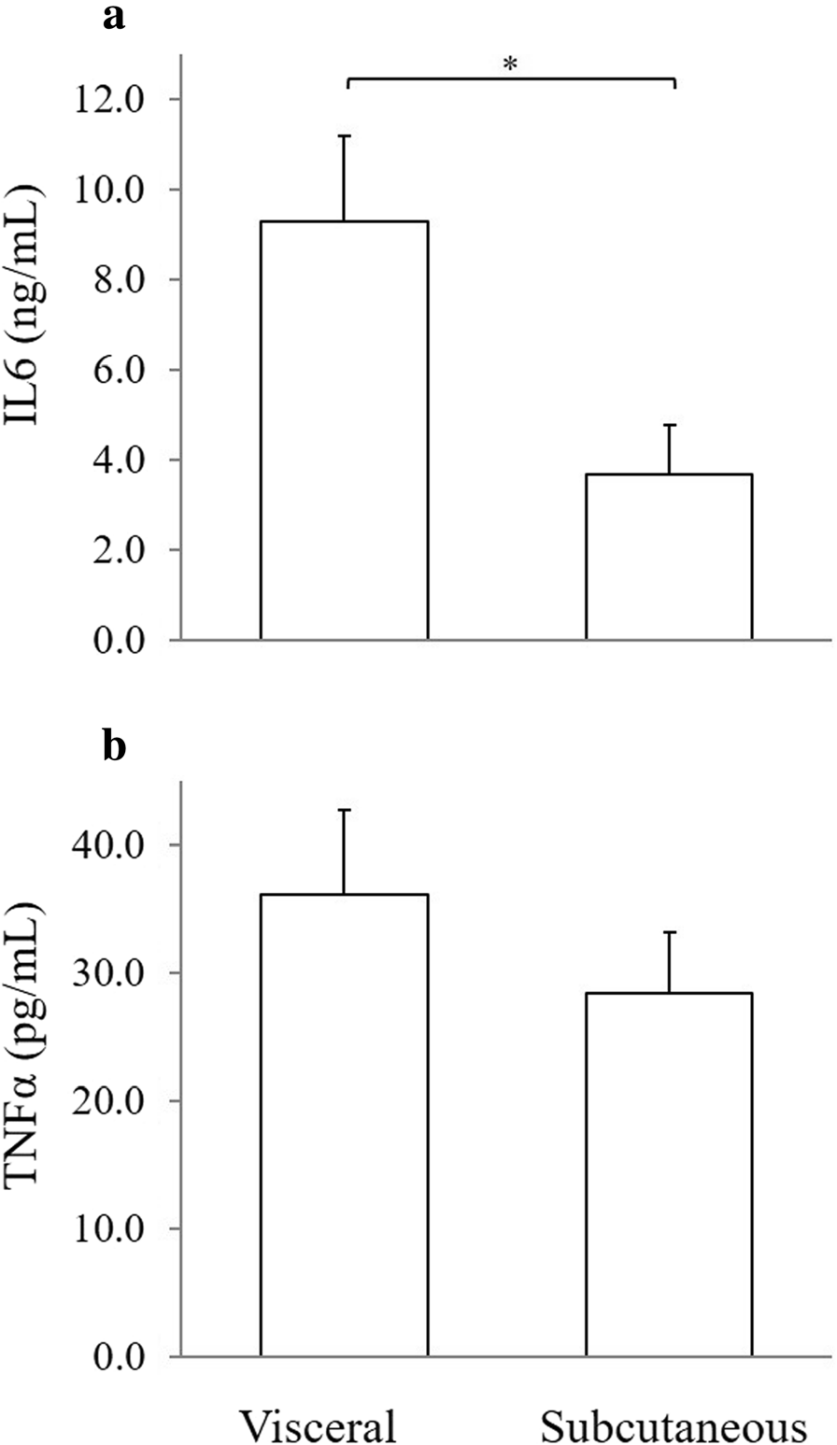
Medium concentrations of IL6 (**a**) and TNFα (**b**) in control stromovascular cells cultures from visceral (*n* = 18) and subcutaneous (*n* = 17) adipose tissue of healthy cats. Bar represents the adjusted mean medium concentration and error bar represents 1 standard error. *P = 0.003

**Fig. 2:**
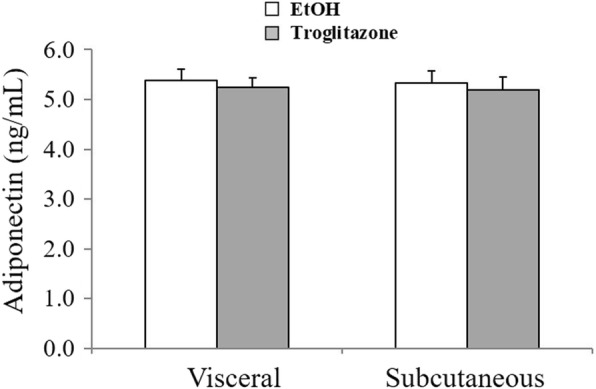
Medium adiponectin concentrations in primary adipocytes control cultures and troglitazone-treated cultures from visceral and subcutaneous adipose tissue of healthy cats. Bar represents the adjusted mean medium concentration and error bar represents 1 standard error. In the visceral depot there were 18 control and 18 troglitazone-treated cultures. In the subcutaneous depot there were 17 control and 17 troglitazone-treated cultures

Fat depot had a significant effect on medium IL6 concentrations; adjusted mean medium IL6 concentration from visceral derived SVC was higher (*P* = 0.003) compared to subcutaneous derived SVC. No significant effect of fat depot was demonstrated on medium concentrations of TNFα (*P* = 0.2) and adiponectin (*P* = 0.8, Figs. 1 and 2).

### Effect of treatment on adipokines

No significant effect on medium adiponectin concentration was observed following troglitazone treatment (*P* = 0.4, Fig. 2) or FA treatments at 25 μM (*P* = 0.2), 50 μM (*P* = 0.8), or 100 μM (*P* = 0.7).

Interaction terms between fat depot and FA treatment were significant at 25, 50 and 100 μM in the analyses of IL6 and TNFα (*P* < 0.05 for all), therefore results of separate analyses for each fat depot are reported.

**Fig. 3:**
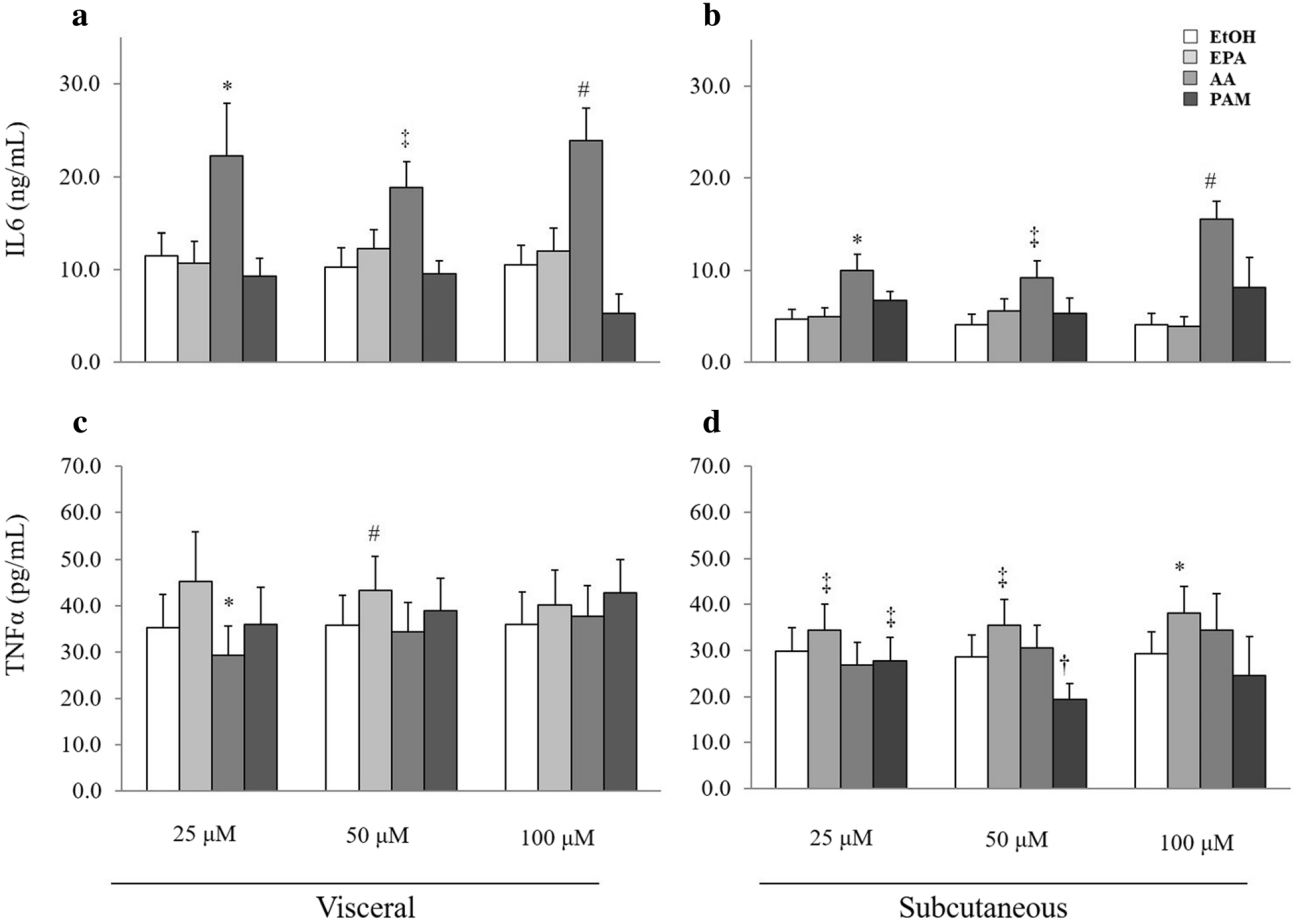
Medium IL6 (**a**, **b**) and TNFα (**c**, **d**) concentrations in stromovascular cells cultures treated with EPA, ARA, PMA or EtOH (control) from visceral (**a**, **c**) and subcutaneous (**b**, **d**) adipose tissue of healthy cats. Bar represents the adjusted mean medium concentration and error bar represents 1 standard error. * *P* < 0.05, † *P* < 0.01, ‡ *P* < 0.005, # *P* < 0.001 compared to control with Bonferroni correction. In the visceral depot there were 18 control cultures. The number of cultures treated with EPA, ARA, and PMA were 18, 17, and 17 at 25 μM, 17, 18, and 18 at 50 μM and 17, 18, and 16 at 100 μM. In the subcutaneous depot there were 17 control cultures. The number of cultures treated with EPA, ARA, and PMA were 17, 17, and 16 at 25 μM, 16, 16, and 17 at 50 μM, and 16, 17, and 17 at 100 μM

FA treatment at 25 μM, 50 μM, and 100 μM had significant effects on medium IL6 concentration both in visceral (*P* = 0.029, *P* < 0.001, and *P* < 0.001, respectively) and subcutaneous (*P* = 0.007, *P* < 0.001, and *P* < 0.001, respectively) derived SVC. ARA treatment was associated with significantly higher medium IL6 concentration than the control at 25 μM, 50 μM, and 100 μM in subcutaneous (*P* = 0.045, *P* = 0.002, and *P* < 0.001, respectively) and visceral (*P* = 0.034, *P* = 0.001, and *P* < 0.001, respectively) derived SVC (Fig. 3a and b).

FA treatment at 25 μM and 50 μM had significant effects on medium TNFα concentrations in visceral (*P* = 0.009 and *P* < 0.001, respectively) and subcutaneous (*P* < 0.001 and *P* < 0.001, respectively) derived SVC. At 100 μM, FA treatment had a significant effect on medium TNFα concentrations in subcutaneous (*P* < 0.001) but not in visceral (*P* = 0.1) derived SVC. ARA treatment at 25 μM was associated with significantly lower medium TNFα concentrations than control in visceral (*P* = 0.032) derived SVC. EPA treatment was associates with significantly higher adjusted medium TNFα concentrations than control in subcutaneous derived SVC at 25 μM, 50 μM, and 100 μM (*P* = 0.002, *P* = 0.001, and *P* = 0.015, respectively) and in visceral derived SVC at 50 μM (*P* < 0.001). PAM treatment was associated with significantly lower medium TNFα concentrations than control in subcutaneous derived SVC at 25 μM and 50 μM (*P* = 0.001 and *P* = 0.008, respectively) (Fig. 3c and d).

## Discussion

Secretion of IL6 from feline adipose tissue SVC was higher in visceral compared to subcutaneous tissue. Although not statistically significant, a similar trend of difference was noted for TNFɑ. These findings are in agreement with previous reports of higher inflammatory cytokines secretion from visceral compared to subcutaneous tissue in humans and dogs [6, 30], but in contrast to findings of a study in cats that demonstrated higher IL6 expression in subcutaneous fat compared to visceral fat, while no difference in TNFα expression was found [31]. Since secretion of inflammatory cytokines increases with rising numbers of macrophages within the adipose tissue, and decreases with higher expression and activation of PPARγ in macrophages [32], the differences in secretion of inflammatory cytokines between the fat depots may result from higher numbers of SVC and lower expression of PPARγ in visceral than in subcutaneous adipose tissue, as was demonstrated in humans [6, 33]. Studies in cats, on the other hand, showed higher expression of PPARγ2 in visceral compared to subcutaneous fat, as well as no difference in the number of macrophages and expression of PPARγ1 between the two fat depots [12, 31]. The higher PPARγ2 expression is consistent with the lower IL6 expression in visceral fat found in that previous study [31], but in contrast to the finding of higher IL6 secretion in the present study. This disparity may result from the different culture and study design. In the previous study, IL6 expression was measured in whole adipose tissue that contains mostly mature adipocytes, whereas in the present study, IL6 expression was measured in the SVC fraction that contains macrophages that are the main source of inflammatory cytokines secretion. Further studies are warranted to clarify the association between expression of inflammatory cytokines and expression of PPARγ and the number of macrophages in different fat depots and their specific fractions in cats.

Secretion of adiponectin from feline mature adipocytes was similar in visceral and subcutaneous adipose tissue depots. This finding is in agreement with a previous study in healthy cats [34], although other studies in cats reported higher adiponectin gene expression in visceral compared to subcutaneous fat [12, 31, 35]. These previous reports in cats are opposite to findings of most studies in humans and animals of lower adiponectin expression and secretion in visceral compared to subcutaneous fat [30, 36]. Adiponectin production is higher in adipocytes of smaller size and with higher level of expression and activation of PPARγ [37]. In cats, adipocytes derived from visceral fat were smaller compared to adipocytes derived from subcutaneous fat [31], and PPARγ expression was found to be higher in visceral compared to subcutaneous fat [12, 35], providing support for the reported higher adiponectin expression in visceral fat in cats. The present study may have been limited to detect a potential difference in adiponectin secretion as no association between its concentration and PAV was found. Small sample size might have also been a limiting factor.

No association between BCS and concentrations of adipokines secreted into the culture medium was found in the present study. These results are in agreement with some previous studies in cats that did not reveal associations between body condition and expression or concentrations of adiponectin [35, 38–40], IL6 [31, 41], or TNFα [42]. In contrast, other studies in cats reported decreased circulating concentrations and adipose tissue mRNA expression of adiponectin [17–19, 31, 34, 41–46] and increased expression of TNFα [20, 31] in obesity. Reduction in adipose tissue PPARγ expression has been demonstrated in obese cats [47], similar to findings in humans and rodent models [33]. In addition, adipocytes were larger in obese compared to lean cats [31]. The numbers of macrophages increase in adipose tissue in obesity in humans [7], while in cats, the number of T-lymphocytes was higher in obese compared to lean cats [31]. These changes are expected to result in decreased production of adiponectin by adipocytes and increased production of IL6 and TNFα by adipose tissue macrophages in obesity. The lack of the expected associations between BCS and medium adipokines concentrations in the present study may be due to the narrow range of body condition scores (5–6/9) among 16 of 18 of the cats, and as a result, the presence of only minimal obesity-related changes affecting adipokines secretion.

ARA had a stimulatory effect on IL6 secretion from adipose tissue SVC both in visceral and in subcutaneous depots, while EPA and PAM did not change IL6 secretion. In line with these findings in cats, ARA was demonstrated to increase secretion of IL6 as well as monocyte chemoattractant protein-1 in adipocytes cell line [48, 49]. This increase was attenuated by EPA and was demonstrated to be mediated through activation of the nuclear transcription factor NF-κB [48]. This pattern of IL6 response to FA in feline adipose tissue may be viewed as consistent with the role of ARA in enhancing immune function [50]. These results in cats are different from our recent findings in canine adipose tissue SVC culture, where IL6 secretion was increased by PAM and decreased by EPA, while no effect of ARA was demonstrated [51], as well as results of studies in macrophages [52–54], adipocytes [55, 56], and endothelial cells [57].

The effect of FA on secretion of TNFα from feline SVC was less consistent among fat depots and FA concentrations, and included increased secretion in response to EPA treatment and decreased secretion in response to ARA and PAM treatments at some FA concentrations in subcutaneous or visceral SVC. These results are in contrast to previous reports that implicated SFA in induction of TNFα in murine and human macrophage cell lines [58] as well as in murine adipocytes cell line [59], while variable PUFA were shown to suppress its induction [52, 53, 60]. The stimulatory effect of EPA on TNFα secretion from feline SVC is surprising in view of the recognized anti-inflammatory effect of this FA. Interestingly, TNFα mRNA expression was increased by dexamethasone in differentiated canine adipocytes [61]. However, the explanation for this finding is unclear. An inhibitory effect of ARA on TNFα secretion was found only at a low FA concentration in visceral fat, and the explanation to this opposite effect of ARA on TNFα compared to IL6 is not clearly apparent. IL6 was previously shown to suppress TNFα production in skeletal muscle [62]. Although the presence of a similar interaction between these cytokines has not been shown in adipose tissue or macrophages, it may provide an explanation for the seemingly opposite effect of ARA on IL6 and TNFα in the present study. The inhibitory effect of PAM on TNFα secretion was present in subcutaneous fat only and was also unexpected. This finding is in contrast to findings in humans adipocytes, where an increase in mRNA expression of TNFα was found in response to PAM [63]. The inconsistent results of TNFα secretion, as well as the lack of association between SVC score and TNFα concentration may support the possibility of other unknown effects on its secretion and question the reliability of these results.

Secretion of adiponectin from mature feline adipocytes was not altered by troglitazone or any of the FA studied (EPA, ARA, or PAM). A potential effect of EPA to increase secretion of adiponectin is thought to be mediated through its interaction with PPARγ [64]. Pharmacological agonists of PPARγ have been shown to increase circulating adiponectin concentrations in cats [14] similar to findings in humans and dogs [15, 16, 65]. The association between serum concentrations of adiponectin and EPA was positive in non-obese cats, but negative in obese cats [66]. Revealing a potential effect of troglitazone and FA on adiponectin secretion may have been hindered by the design of the present study. Secretion of adiponectin was evaluated in the first 2 days of culture only; therefore potential effects of troglitazone or FA following a longer time period in culture may be possible, as demonstrated previously [67]. In addition, the lack of association between adiponectin secretion and the volume of cultured adipocytes (PAV) also suggests that a potential effect on adiponectin was not reflected by measuring its secretion and evaluation of adiponectin gene expression could have provided additional information.

This study has several additional limitations, including the lack of detailed metabolic evaluation of the cats including serum concentrations of glucose, lipids, and adipokines, and lack of direct determination of cell viability and adipocytes size. Nevertheless, the significant positive association between SVC score and IL6 concentrations provides indirect evidence for the reliability of the culture system for evaluation of IL6 secretion, while the lack of association between PAV and adiponectin concentrations or SVC score and TNFα concentrations suggests that this study might have been limited by its design to reveal potential and consistent effects on adiponectin and TNFα secretion and further study is warranted. In addition, since only intact female cats were included in the study, the effect of gender on adipokines secretion could not be evaluated.

## Conclusions

The differential secretion of IL6 by fat depots in cats, with increased secretion of IL6 from visceral compared to subcutaneous adipose tissue SVC, suggests that visceral fat may be the primary contributor of adipose tissue origin to systemic inflammation. Since IL6 secretion was not associated with body condition, further investigation is required to determine whether visceral obesity in cats poses increased risk for metabolic disorders, such as type-2 diabetes and hepatic lipidosis, similar to findings in human subjects. ARA was demonstrated to have a stimulatory effect on secretion of IL6 from feline adipose tissue, while no effect of EPA was demonstrated. A potential anti-inflammatory effect of fish oil may be mediated by substitution of ARA by EPA and a consequent reduction in IL6 secretion. Further studies are warranted to elucidate potential mechanisms.

## Methods

### Cats

Eighteen client-owned healthy intact female cats admitted to the Veterinary Teaching Hospital at Michigan State University for routine ovariohysterectomy were included in the study. Cats were determined to be healthy based on physical examination, absence of clinical signs indicating disease, and no abnormalities on a presurgical minimal data base including a complete blood count, kidney function, and liver enzymes. Body condition was evaluated using a body condition scoring (BCS) system on a 1–9 scale [68].

### Adipose tissue preparation and digestion

At the time of ovariohysterectomy procedure, omental visceral adipose tissue samples were obtained from all cats (*n* = 18) and subcutaneous adipose tissue samples were obtained from cats that had sufficient fat directly under the skin incision (*n* = 17). The samples (0.5–2.0 g) were obtained aseptically. Each sample was immediately placed in 15 mL of fresh cell Dulbecco’s modified Eagle’s medium/Ham’s F12 medium (1:1) supplemented with 10 mM HEPES, 10 mg/mL fatty acid free bovine serum albumin, 5 μg/mL ethanolamine, 0.1 ng/mL sodium selenite, 55 μmol/L ascorbic acid, 200 nmol/L adenosine, 100 U/mL penicillin, 0.1 mg/mL streptomycin, and adjusted to a pH of 7.4 with approximately 1.2 g/mL sodium bicarbonate at 4 °C. Samples were transferred to the laboratory for processing within 30 min.

Each tissue sample was washed with Hanks’ Balanced Salt Solution (HBSS, Sigma H8265) to remove large blood clots; blood vessels and connective tissue were excised, and the remaining tissue was minced into 1–5 mg fragments. The minced tissue was incubated in medium at room temperature for 30–60 min to remove soluble factors and then washed with Hanks’ Balanced Salt Solution.

To separate mature adipocytes from SVC, tissue fragments of each tissue sample were separately immersed in cell culture medium (2 mL/g tissue) that had been supplemented with collagenase (C6885, Sigma-Aldrich; 1 mg/mL) and digested for 60 min on a rotating platform (100 rotations/min) at 38 °C. After incubation, the digest was passed through a 100 μm nylon mesh and centrifuged (450Xg, 22 °C) for 5 min. The mature adipocytes in the supernatant were separated from the pelleted SVC by aspiration. The cell pellet was treated with red blood cell lysing buffer (1 mL/g digested tissue) for 3 min. Then, the mature adipocytes and SVC were separately washed in cell culture medium and centrifuged (450Xg, 22 °C for 5 min) 3 times. For each tissue sample, aliquots of 100 μL adipocytes were each suspended in 1 mL fresh medium in 5 mL polypropylene tubes. SVC from each tissue sample were suspended in fresh medium at a final concentration of approximately 100,000 cells/mL and seeded into a 24-well plate at 1 mL per well. Adipocytes and SVC were incubated in a humidified incubator with 5% CO_2_ at 38 °C.

### Incubation and treatment

Fatty acids (EPA: U-99-A, ARA: U-71-A, PAM: N-16-A; Nu-Chek Prep, Inc) stock solutions (50 mM) were prepared in absolute ethanol and stored at -20C in glass tubes protected from light. FA treatment media were prepared by dilution of stock solutions of fatty acids in cell culture medium to a final concentration of 25 μM, 50 μM, or 100 μM. Troglitazone (Sigma, T2573) treatment medium was prepared by dilution into a final concentration of 10 μM. Control medium was prepared by dilution of the same volume of ethanol in cell culture medium. The final concentration of ethanol was < 0.2%. Treatment and control media were then incubated at 38 °C for 2 h prior to being added to the cells.

After 24 h of initial incubation, medium from all tubes (cultured mature adipocytes) and wells (cultured SVC) was aspirated. Then, either fresh control medium or treatment medium (troglitazone at 10 μM, or EPA, ARA, or PAM, at 25 μM, 50 μM, or 100 μM) was added to each tube or well. Following 48 h of incubation, medium from each tube and well was collected and centrifuged (15,000Xg, 4 °C) for 10 min; the supernatant was stored at − 80 °C until analysis. Prior to medium collection, small aliquots of medium containing adipocytes were aspirated from each tube into capillary hematocrit tubes and centrifuged (15,000Xg, 24 °C) for 1 min in a microhematocrit centrifuge to measure the fractional occupation of the suspension by the adipocytes (packed adipocytes volume -PAV) [69]. Tubes with oil drops, indicating cell death, or PAV of 1% or less were excluded from further analysis. Culture plates were examined microscopically and wells were scored according to the percentage of well area occupied by SVC (SVC score; 1 = 10–20%, 2 = 21–40%, 3 = 41–60%, 4 = 61–80, 5 = 81–100%). Wells with detached cells or less than 10% area of occupation were excluded from further analysis.

### Medium analysis

Samples of medium from cultured mature adipocytes were analyzed to determine concentrations of adiponectin and samples of medium from cultured SVC were analyzed to determine concentrations of IL6 and TNFα. Medium concentrations of adiponectin, IL6 and TNFα were measured using commercially available assays (Mouse/rat Adiponectin ELISA, B-Bridge; Feline IL6 ELISA, R&D Systems; Feline TNFα ELISA, R&D Systems). Medium samples were diluted 1:100 in fresh medium in preparation for the IL6 assay. The dynamic ranges of the assays were 0.016–8 ng/mL for adiponectin, 6.1–2000 pg/mL in diluted samples for IL6, and 2.4–1000 pg/mL for TNFα. Maximal intra- and inter-assay coefficients of variation were 5 and 7% for adiponectin, 3 and 7% for IL6, and 8 and 10% for TNFα.

### Data analysis

Associations between fat depot (visceral or subcutaneous) or BCS (1 to 9) and each outcome variable (medium concentrations of adiponectin, IL6, or TNFα) were evaluated using generalized estimating equations (GEE). Troglitazone treatment (control, troglitazone) was added as a factor in the analysis of adiponectin. At each FA concentration (25 μM, 50 μM, and 100 μM), associations between FA treatment (control, EPA, ARA, PAM) and the same outcome variables were evaluated using GEE with fat depot as an additional factor and BCS and as an additional covariate. In all GEE models, PAV was entered as an additional covariate in the analysis of adiponectin and SVC score was entered as an additional covariate for IL6 and TNFα in order to control for the potential differences in numbers of adipocytes or SVC among test tubes or wells, respectively. Fat depot and treatment were set as within-subject variables. Interaction effects between fat depot and treatment or BCS were assessed. Non-significant interaction terms were removed from the models. Separate GEE models were analyzed and results were reported separately for each fat depot in analyses in which a significant (*P* < 0.05) interaction was observed. The residuals were normally distributed in all models. Bonferroni correction was applied for multiple post-hoc comparisons between different treatments and the control. Data were analyzed using a commercially available statistic program (SPSS® 22.0 for Windows). *P* ≤ 0.05 was considered statistically significant. Results of the GEE analyses are presented as means (95% confidence interval) adjusted for the additional factors and covariates included in each analysis.

## Data Availability

The datasets used and/or analysed during the current study are available from the corresponding author on reasonable request.

## Abbreviations

ARA: Arachidonic acid (20:4n-6)
BCS: Body condition score
DHA: Docosahexaenoic acid (22:6n-3)
EPA: Eicosapentaenoic acid (20:5n-3)
FA: Fatty acids
GEE: Generalized estimating eqs.
IL: Interleukin
n3PUFA: n3 polyunsaturated fatty acids
PAM: Palmitic acid (16:0)
PPAR: Peroxisome proliferator-activated receptor
SVC: Stromovascular cells
TNF: Tumor necrosis factor
